# Effects of contrast-enhancement, reconstruction slice thickness and convolution kernel on the diagnostic performance of radiomics signature in solitary pulmonary nodule

**DOI:** 10.1038/srep34921

**Published:** 2016-10-10

**Authors:** Lan He, Yanqi Huang, Zelan Ma, Cuishan Liang, Changhong Liang, Zaiyi Liu

**Affiliations:** 1 Department of Radiology, Guangdong General Hospital, Guangdong Academy of Medical Sciences, Guangzhou, Guangdong, 510080, China; 2 School of Medicine, South China University of Technology, Guangzhou, Guangdong, 510006, China

## Abstract

The Effects of contrast-enhancement, reconstruction slice thickness and convolution kernel on the diagnostic performance of radiomics signature in solitary pulmonary nodule (SPN) remains unclear. 240 patients with SPNs (malignant, n = 180; benign, n = 60) underwent non-contrast CT (NECT) and contrast-enhanced CT (CECT) which were reconstructed with different slice thickness and convolution kernel. 150 radiomics features were extracted separately from each set of CT and diagnostic performance of each feature were assessed. After feature selection and radiomics signature construction, diagnostic performance of radiomics signature for discriminating benign and malignant SPN was also assessed with respect to the discrimination and classification and compared with net reclassification improvement (NRI). Our results showed NECT-based radiomics signature demonstrated better discrimination and classification capability than CECT in both primary (AUC: 0.862 vs. 0.829, p = 0.032; NRI = 0.578) and validation cohort (AUC: 0.750 vs. 0.735, p = 0.014; NRI = 0.023). Thin-slice (1.25 mm) CT-based radiomics signature had better diagnostic performance than thick-slice CT (5 mm) in both primary (AUC: 0.862 vs. 0.785, *p* = 0.015; NRI = 0.867) and validation cohort (AUC: 0.750 vs. 0.725, *p* = 0.025; NRI = 0.467). Standard convolution kernel-based radiomics signature had better diagnostic performance than lung convolution kernel-based CT in both primary (AUC: 0.785 vs. 0.770, *p* = 0.015; NRI = 0.156) and validation cohort (AUC: 0.725 vs.0.686, *p* = 0.039; NRI = 0.467). Therefore, this study indicates that the contrast-enhancement, reconstruction slice thickness and convolution kernel can affect the diagnostic performance of radiomics signature in SPN, of which non-contrast, thin-slice and standard convolution kernel-based CT is more informative.

Solitary pulmonary nodule (SPN) is defined as approximately rounded opacity measuring up to 3 cm in diameter appears on imaging as focal opacities surrounded by aerated lung^1^,^2^. Differential diagnosis of SPN ranges from the primary lung cancer to various benign lesions, such as hamartoma^3^,^4^. However, 20–50% of SPNs are found as early sign of lung cancer^5^, which remain the leading cause of cancer related incidence and mortality worldwide^6^,^7^. Therefore, accurate differential diagnosis of SPN can reduce cancer related mortality by characterizing malignant tumors and thus spare patients with benign disease from undergoing unnecessary surgery.

Computed tomography (CT) as a widely available noninvasive modality for detecting lung cancer in clinical practice, has an important role in the differential diagnosis of SPN^8^,^9^,^10^,^11^. However, conventional visual assessment of SPN on CT, such as tumor size, density and margins, has an lower diagnostic accuracy of approximately 60% in differentiating benign from malignant SPN^12^. Unlike the traditional interpretation of CT images, “Radiomics”, which is the practice of processing high-throughput extraction of quantitative features to convert images into mineable data for decision support^13^, has been proposed to noninvasively decode tumor phenotype^14^,^15^,^16^. Among the diagnostic objective features of SPN, CT-based texture analysis could effectively differentiate benign from malignant lesions^17^,^18^. However, a recent study by Dennis *et al*. indicated that the inter-scanner differences existing among different CT scanner could affect the variability in the values of radiomics features^19^. Being the most common varied factors in clinical settings on the imaging modality, whether the imaging acquisition parameters of contrast-enhancement, reconstruction slice thickness and convolution kernel could affect the diagnostic performance of radiomics features on the differential diagnosis of SPN is an interesting field that has been explorated^13^,^20^. Although individual CT texture feature is useful in the characterization of SPN^17^,^18^,^21^, integrating multiple features into a predictive panel as a radiomics signature may be a robust approach for quantifying tumor phenotype^22^,^23^. Thus, regarding the influence of scanning parameters on the individual feature performance in the previous studies^13^,^19^,^24^, radiomics signature could consequently make impact on the performance of differential diagnosis of SPN.

Therefore, the purpose of this study was to investigate the effects of contrast-enhancement, reconstruction slice thickness and convolution kernel on the differential diagnosis performance of radiomics signature in SPN, and to determine the optimal imaging parameters (contrast-enhancement, reconstruction slice thickness and convolution kernel) for extracting radiomics features.

## Methods

### Patients

The retrospective study was approved by the Research Ethics Committee of Guangdong General Hospital, Guangdong Academy of Medical Sciences (protocol No. GDREC2015192H). Due to the retrospective nature of the study, our institutional review board approved the review of patient data before its commencement and waived the requirement for informed consent. The institutional database was evaluated to collect a primary cohort of this study from January 2010 to December 2012. Patients with biopsy- or surgery-proven primary lung malignancy or benign lesions were enrolled. From January 2013 to July 2015, patients who met the same criteria were included to form an independent validation cohort. Baseline clinical data including age and gender were recorded, and the dates of baseline CT imaging were also recorded.

### CT Image Acquisition

All patients underwent non-contrast and contrast-enhanced CT with a multi-detector row CT (GE Light-speed Ultra 8; GE Healthcare, Hino, Japan). Contrast-enhanced CT image was performed after 25 s delay following intravenous administration of 85 ml of iodinated contrast material (Ultravist 370, Bayer Schering Pharma, Berlin, Germany) at a rate of 3.0 ml/s with a pump injector (Ulrich CT Plus 150, Ulrich Medical, Ulm, Germany) after routine non-contrast CT. The fixed acquisition parameters were as follows: 120 kV; 160 mAs; 0.5- or 0.4-second rotation time; detector collimation: 8 × 2.5 mm or 64 × 0.625 mm; field of view, 350 × 350 mm; matrix, 512 × 512. Each patient of the study had four sets of chest CT images with different imaging parameters of contrast-enhancement parameter (non-contrast or contrast-enhancement), reconstruction slice thickness (5 mm or 1.25 mm) and convolution kernel (standard or lung), which were separately labeled as group 1 (non-contrast + 1.25 mm + standard convolution kernel), group 2 (contrast enhancement + 1.25 mm + standard convolution kernel), group 3 (non-contrast + 5 mm + standard convolution kernel) and group 4 (non-contrast + 5 mm + lung convolution kernel). Generation of CT images utilizing different convolution kernels can optimize lesion detection. Lung convolution kernel is generated when high-pass filter algorithm is used, with high spatial frequencies and noise preserved; while low-pass algorithm enables the generation of standard kernel image, with high spatial frequency contribution and noise decreased. The lung convolution kernel and standard convolution kernel are dependent on the vendor of GE.

### Radiomics feature extraction

**Figure 1:**
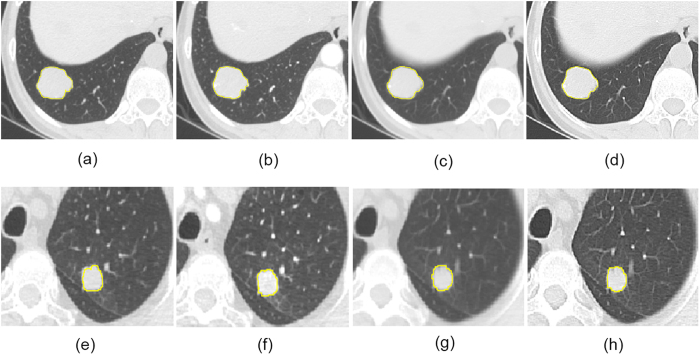
The delineations of ROI for 2 patients who have benign tumor (hamartoma) in group 1 (**a**), group 2 (**b**), group 3 (**c**) and group 4 (**d**), and malignant tumor (adenocarcinoma) in group 1 (**e**), group 2 (**f**), group 3 (**g**) and group 4 (**h**).

All 4 sets of the CT images were used for radiomics feature extraction, after retrieved from the picture archiving and communication system (PACS) (Carestream, Canada). In-house feature extraction algorithm was implemented in Matlab 2014a (Mathworks, Natick, USA). In total, 150 radiomics features which covered the category of gray-level histogram and gray-level co-occurrence matrix (GLCM) were extracted from each set of CT image. A region of interest (ROI) was delineated initially around the tumor outline for the largest cross-sectional area on each set of CT images. Figure 1 demonstrates the ROI delineations for 2 patients who have malignant and benign tumor respectively, with their size and diameter being listed Supplementary Table S1, respectively. The ROI was further refined by excluding air area with a threshold that removed from analysis any pixels with attenuation values below −50 HU and beyond 300 HU. A Laplacian of Gaussian spatial band-pass filter (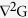) was used to derive image features at different spatial scales by turning the filter parameter between 0 and 2.5 (0, 1.0, 1.5, 2.0, 2.5). The Laplacian of Gaussian filter (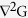) distribution is given by


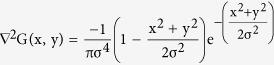


x, y denote the spatial coordinates of the pixel and σ is the value of filter parameter.

The feature extraction algorithms are described in Supplementary Method S1, and series of gray-level histogram and gray-level co-occurrence matrix (GLCM) features derived were also listed in Table 1.

### Intra-reader reproducibility of radiomics features

Intra-reader reproducibility of radiomics feature extraction was initially analyzed with 40 randomly chosen patients (30 malignant and 10 benign) for ROI delineation. The same radiologist who has 10 years’ experience in chest CT interpretation repeated the generation of radiomics features twice in a 1-week period followed the same procedure. The size and diameter of each delineated ROI were measured and recorded. Intra-class correlation coefficients (ICCs) were used to evaluate the intra-reader agreement of the size and diameter of the tumor and each of the 150 radiomics features extracted from the delineated ROIs with a value greater than 0.75 indicating good intra-reader agreements^25^.

### Statistical analysis

All statistical analysis in this study was conducted with R software, version 3.2.1 (http://www.Rproject.org).

Differences in age, gender of patients between benign and malignant in both the primary and validation cohort were compared by using the independent sample *t* test or the Mann-Whitney U test, the Chi-Squared test or the Fisher exact test, where appropriate. And the same tests were also applicable for the assessment of difference in patients’ age, gender between primary and validation cohort.

### Diagnostic performance of radiomics features

The association of the radiomics features on discrimination between benign and malignant SPN in both primary cohort and validation cohort across different sets of CT images was assessed using Mann-Whitney U test due to its non-normal distribution. Then, the diagnostic performance of the radiomics features was assessed with respect to the area under the curve (AUC) of the receiver operating characteristic curve (ROC). An AUC of 1 indicates perfect discrimination, and random guess gives an AUC of 0.5.

### Feature selection and radiomics signature building

Based on the results of univariate analysis of radiomics features, feature selection and data dimension reduction were done using least absolute shrinkage and selection operator method (LASSO) logistic regression model^26^ to select the most useful prognostic features of all the associated radiomics features identified with the primary cohort. The LASSO, which is suitable for the regression of high dimensional data using the “glmnet” package in R software, is a penalized estimation technique in which the estimated regression coefficients are constrained so that the sum of their scaled absolute values falls below some constant k chosen by cross-validation. This kind of constraint forces some regression coefficient estimates to be exactly zero, thus achieving variable selection while shrinking the remaining coefficients toward zero to reflect the overfitting caused by data-based model selection. The radiomics signature was built for each patient in both the primary and the validation cohort through the linear combination of features selected by their respective coefficients, with a radiomics score calculated for each patients. A larger score indicates a higher probability to be malignant.

### Diagnostic performance and comparison of radiomics signature derived from different CT sets

The potential association of radiomics signature on discrimination between benign and malignant SPN was also assess using Mann-Whitney U test. The diagnostic performance of radiomics signature was assessed in terms of discrimination and classification. ROC curves for each group dataset were constructed and the area under the curves (AUC) were calculated with histopathological diagnosis of SPNs as outcome. Sensitivity, specificity, and accuracy were also derived as the methods of classification measurement.

For the comparison of discrimination ability for radiomics signatures on diagnostic performance in SPN, the nonparametric test of Delong test was used for comparing the difference in AUC of ROC between groups^27^. A two-sided P value less than 0.05 was considered to indicate the statistical significant difference. A net reclassification improvement (NRI) calculation which is regarded as an increasingly popular measure for evaluating improvements in risk predictions^28^,^29^,^30^ was also applied for assessing whether one group of prediction performance is better than another. The formula for calculating the NRI (Net Reclassification Index):


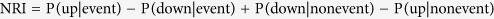


In this formula, upward movement (up) was defined as a change into higher category based on the new biomarker and downward movement (down) as a change in the opposite direction. The value of NRI can either be positive or negative. A positive value of NRI derived in this study indicates a net improvement in risk classification for patients with SPN.

Finally, the same comparison for each group of radiomics signatures was assessed in the independent validation cohort.

## Results

### Clinical characteristics and distribution of patients

In total, we retrospectively identified 240 consecutive patients with SPN (benign, n = 60, such as hamartoma (33), pulmonary crytococcosis (5), inflammatory pseudotumor (5), inflammatory granuloma (10), pulmonary sclerosing hemangioma (7); malignant, n = 180, such as lymphoepithelioma (6), squamous-cell carcinoma (22), adenocarcinoma (145), metastatic tumor (7)) between January 2010 and July 2015 who underwent chest CT as the whole study cohort. 120 cases of the institutional database from January 2010 to December 2012 were identified as the primary cohort and other 120 cases from January 2013 to July 2015 were identified as the validation cohort, respectively. Distribution of patients’ characteristics in both primary and validation cohorts between the benign group and malignant group are presented in Table 2. The patients in benign group were younger than that in malignant group (p < 0.001 for both primary and validation cohort), but there was no significant difference in gender between benign and malignant group in both primary (p = 0.745) and validation cohort (p = 0.832). No difference was found between the primary and the validation cohort in the clinical characteristics (p = 0.253 for age, and p = 0.514 for gender).

### Intra-reader reproducibility of radiomics features

Satisfactory intra-reader reproducibility of ROI delineation for the 4 groups was achieved, with an intra-class correlation coefficient (ICC) of 0.795 and 0.802 for the size and diameter, respectively. The ICCs for 150 radiomics features had been listed in the Supplement Table S2, ranged from 0.752 to 1.000.

### Diagnostic performance of radiomics features

**Figure 2:**
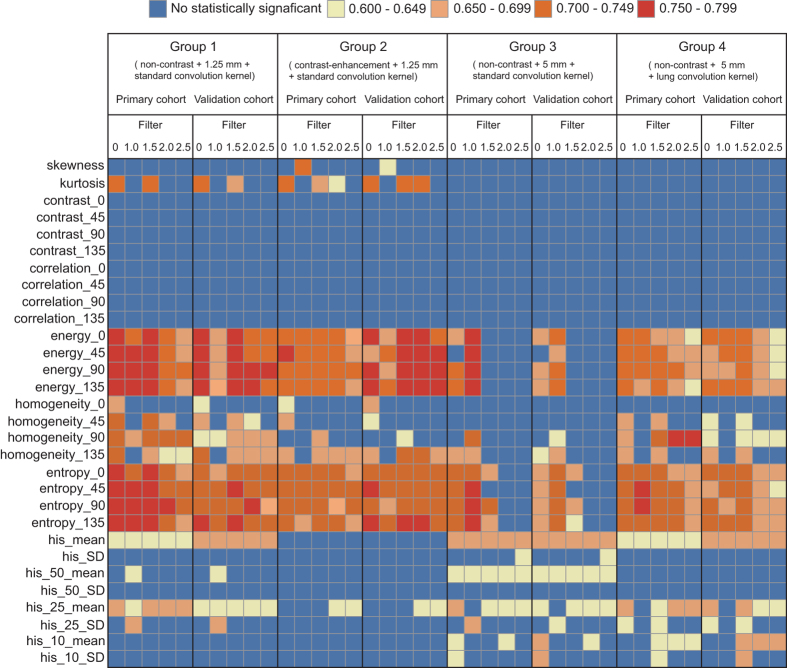
Color mapping for the univariate analysis for each of radiomics features on discrimination between benign and malignant SPNs in both primary cohort and validation cohort across different groups. The y-axis presents the categories of radiomics features; the x-axis presents the different filter values for the features extraction in both primary and validation cohort across the different groups. Blue cell represents the feature showed no significant association with the status of SPN, while other color cells represent the features showed significant association, with different ranges of AUC derived.

There were 66, 52, 39 and 62 features which showed significant association between the radiomics features and the status of SPN in group 1, group 2, group 3 and group 4, respectively (*p* < 0.05) (Fig. 2). The univariate analysis between each of the significant associated radiomics features were presented in Fig. 2 and listed in Supplementary Table 3. As shown in Fig. 2, the number of the features showing significant association with status of SPN derived from group 1 was the largest compared to other groups. Furthermore, the number of features showing good diagnostic performance (AUC > 0.75) was also the largest.

### Feature selection and radiomics signature building

There were 12, 4, 3 and 3 features with non-zero coefficients in the LASSO logistic regression model selected in group 1, group 2, group 3 and group 4, respectively (Supplementary Figure S1–S4). These selected features and their individual coefficients were displayed in the form of histogram in Supplementary Figure S5 and listed in Supplementary Table S4. The corresponding radiomics signature score calculation formula was presented in the Supplementary Equations S1–S4. The number of selected features varied greatly among 4 groups. In addition, the categories of selected features also varied across different radiomics signatures.

### Diagnostic performance of radiomics signature derived from different groups

**Figure 3:**
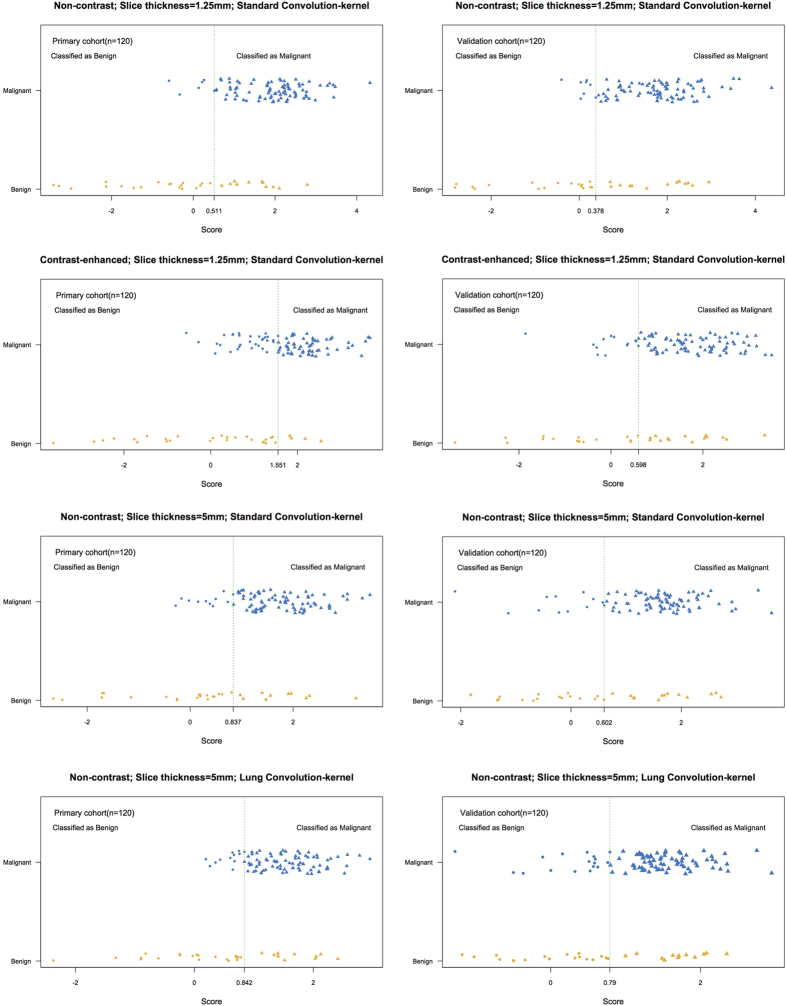
Distributions of score for the radiomics signature on classification SPN status in the primary and validation cohort in different sets of CT imaging (group 1 to group 4). Y axis indicated the true categories of SPNs and X axis indicted the scores of radiomics signatures, which can be used for predicting the categories of SPNs in each group with the best cutoff. The green dotted vertical lines were drawn for the best cutoff on each group.

There was significant difference in radiomics signature scores between benign and malignant patients for four groups in primary cohort (*p* < 0.001), which was consistent with the validation cohort (p < 0.001). Malignant patients generally had higher scores in both the primary cohort and validation cohort (Table 2). The distribution of radiomics signature scores for classification of SPN status in the primary cohort and validation cohort are shown in Fig. 3. Further, the diagnostic performance of radiomics signature varied greatly in both the primary and validation cohort across all 4 groups with a varied AUC of 0.686–0.862, sensitivity of 0.667–0.944, specificity of 0.533–0.867 and accuracy of 0.708–0.858 (Table 3).

### Comparison of diagnostic performance of radiomics signatures derived from different groups

As listed in Table 3, there was significant variability in the diagnostic performance of radiomics signatures in SPN based on features extracted from CT images acquired with different parameters (contrast-enhancement, reconstruction slice thickness and convolution kernel). Although AUCs were different between groups in both primary cohort and validation cohort, the NRIs were also analyzed (Table 4). Our results showed NECT-based radiomics signature demonstrated better discrimination and classification capability than CECT in both primary (AUC: 0.862 vs. 0.829, *p* = 0.032; NRI = 0.578) and validation cohort (AUC: 0.750 vs. 0.735, *p* = 0.014; NRI = 0.023). Thin-slice (1.25 mm) CT-based radiomics signature had better diagnostic performance than thick-slice CT (5 mm) in both primary (AUC: 0.862 vs. 0.785, *p* = 0.015; NRI = 0.867) and validation cohort (AUC: 0.750 vs. 0.725, *p* = 0.025; NRI = 0.467). Standard convolution kernel-based radiomics signature had better diagnostic performance than lung convolution kernel-based CT in both primary (AUC: 0.785 vs. 0.770, *p* = 0.015; NRI = 0.156) and validation cohort (AUC: 0.725 vs. 0.686, *p* = 0.039; NRI = 0.467).

## Discussion

This study demonstrated that incorporating individual radiomics features extracted from CT images as a radiomics signature facilitated the differential diagnosis of SPN, and the variability of acquisition parameters (contrast-enhancement, reconstruction slice thickness and convolution kernel) had effects on the diagnostic performance of radiomics signature in SPN. In addition, we also demonstrated that radiomics signature based on CT images acquired with non-contrast, thin-slice and standard convolution kernel had better performance on the differential diagnosis of SPN.

As one processes of radiomics studies, optimum protocols for image acquisition and reconstruction algorithm have to be identified and harmonized^13^,^20^. The variation caused by different parameters implies that it should be possible to make consistency for this acquisition parameters used in radiomics studies^13^,^19^. To date, most studies of radiomics features have focused on finding robustness features^19^,^31^,^32^,^33^. Among the above studies, Leijenaar *et al*. studied the stability of radiomics features^32^, and Hunter *et al*. identified the high quality machine-robust image features^33^. Although robustness radiomics features were presented in the above previous study, it’s worthy of notice that the impact of acquisition parameters on radiomics signatures could vary widely, which has never been investigated. As expected, our study showed that the contrast-enhancement, reconstruction slice thickness and convolution kernel affected the diagnostic performance of radiomics features as revealed by the univariate analysis, and as well as the corresponding radiomics signatures constructed by LASSO regression method in SPN.

Radiomics signature built based on the non-contrast CT images showed better performance in the differential diagnosis of SPN, compared with the contrast-enhanced CT images. The underlying reason for the better performance on non-contrast images may be that the biological heterogeneity within the tumor that can be depicted by radiomics features may be confounded by the intravenous injected contrast material, which may then result in poorer discrimination between malignant and benign tumors due to the existing intratumoral contrast material^34^,^35^,^36^.

Regarding the reconstruction slice thickness of CT images, the radiomics signature built based on thin slice thickness (1.25 mm) was found to have better performance in the differential diagnosis of SPN as compared with that built on thick slice thickness (5 mm) in our study. Similarly, previous studies had presented that slice thickness could significantly affect the quantification of CT image features, and illustrated that slice thickness of 1.25 mm and 2.5 mm were better than 5 mm for texture features^37^. The underlying reason for the better performance of thin-slice images may be that thicker slice images introduce larger partial pixel artifacts as compared to thinner slice images^37^,^38^.

Furthermore, we found that radiomics signature built based on standard convolution kernel CT had better diagnostic performance than that built based on lung convolution kernel CT. The underlying reason for the better performance on standard convolution kernel images may be that generation of CT images utilizing different convolution kernels can optimize lesion detection. Lung convolution kernel is generated when high-pass filter algorithm is used, with high spatial frequencies and noise preserved; while low-pass algorithm enables the generation of standard kernel image, with high spatial frequency contribution and noise decreased, and work best for tissues with inherently lower contrast, such as lung tissues^39^.

As discussed above, the acquisition parameters (contrast-enhancement, reconstruction slice thickness and convolution kernel) affected the features selection (12, 4, 3 and 3 features selected out of the significant associated radiomics features in group 1, group 2, group 3 and group 4, respectively), with which the corresponding radiomics signature was constructed. Accordingly, the variability of radiomics signature demonstrates different diagnostic performance in SPN. Our results showed that radiomics signature based on the non-contrast, thin-slice and standard convolution kernel-based CT was more informative on differential diagnosis of SPN.

Limitation of this study includes the fact that the variability in radiomics signature on differential diagnosis of SPN could be caused by different types of CT scanners. All sets of images in our study were generated by the same CT scanner. Among the previous study, Dennis *et al*. found that the inter-scanner differences on the variability in the values of radiomics features should be considered^19^. Therefore, there might be an interesting attempt to explore the effects of different CT inter-scanners on the differential diagnostic performance of radiomics signature on differential diagnosis of SPN in future studies. Another limitation of this study includes the fact that the dataset may be skewed, with which 180 patients with malignant cancer and only 60 patients with benign tumor composed the whole study cohort. However, our study consists of all consecutive solitary pulmonary nodules that were biopsy- or surgery-proven malignant or benign nodules in our institution collected from January 2010 to July 2015. All images were collected from patients scanned with the same scanner, since there is variability in the quality and repeatability of radiomics features between CT scanners observed in a previous study^19^. Although the dataset was skewed with limited benign cases, the incidence ratio between benign and malignant cases was representative of the intended population in clinical practice. As noted in the TRIPOD statement (Transparent Reporting of a multivariable prediction model for Individual Prognosis Or Diagnosis)^40^, selectively choosing or omitting participants may cast doubt on the representativeness of the sample to the population in which the marker or model is to be applied and affect its generalizability. So despite that ideally the inconsistence should be minimized to reduce the impact on the comparison of the radiomics signature performance, our study enrolled all consecutive patients eligible to the generalizability.

In conclusion, this study presents that the contrast-enhancement, reconstruction slice thickness and convolution kernel can affect the diagnostic performance of radiomics signature in SPN, of which non-contrast, thin-slice and standard convolution kernel-based CT is more informative. The impact of different CT image acquisition parameters on the performance of radiomics signatures should be considered in the future radiomics studies in SPN.

## Additional Information

**How to cite this article**: He, L. *et al*. Effects of contrast-enhancement, reconstruction slice thickness and convolution kernel on the diagnostic performance of radiomics signature in solitary pulmonary nodule. *Sci. Rep.* **6**, 34921; doi: 10.1038/srep34921 (2016).

## Supplementary Material

Supplementary Information

## Figures and Tables

**Table 1:** Feature extraction algorithms and lists of features derived.

Description	Calculation formula	Feature derived
Gray-level histogram		
***Mean*** measures the average value of the histogram	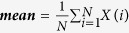	his_mean_σ
***SD*** measures the stability of the gray level histogram	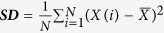	his_SD_σ
***Percentile mean*** **and** ***SD*** measures are calculated from the top 50%, 25%, and 10% of the histogram curve	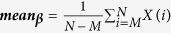	his_β_mean_σ
	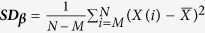	his_SD_β_σ
***Kurtosis*** describes the sharpness of the histogram	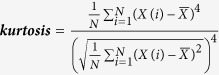	kurtosis_σ
***Skewness*** describes the degree of asymmetry around the mean value in the gray level histogram	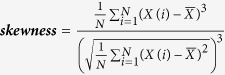	skewness_σ
Gray-Level Co-Occurrence Matrix (GLCM)		
***Contrast*** measures local intensity variation, reflects the uniformity of image grayscale distribution and the degree of thickness in texture	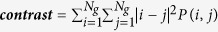	contrast_α_σ
***Correlation*** measures the gray level linear dependence between the pixels at the specified positions relative to each other	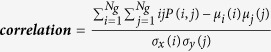	correlation_α_σ
***Entropy*** describes the inhomogeneity of an image	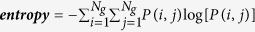	entropy_α_σ
***Energy*** is the sum of squares of entries in the GLCM, measures the image homogeneity	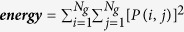	energy_α_σ
***Homogeneity*** weights as the inverse of the Contrast weight	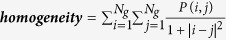	homogeneity_α_σ

Note: *X*(*i*) indicates the intensity of gray level i; N denotes the sum of pixels in the image; β indicates the top percentage of the histogram curve, which could be 50%, 25%, and 10%; M denotes the number of pixels in the histogram on the percentage of (1 − β); *x, y* denote the spatial coordinates of the pixel; P(*i, j*) is the co-occurrence matrix by the δ = 1 and θ(0°, 45°, 90°, 135°); N_g_ denotes the number of discrete intensity levels in the image; *μ* is the mean of P(*i, j*); *μ*_x_(*i*) is the mean of *P*_*x*_(*i*); *μ*_y_(*j*) is the mean of *P*_*y*_(*j*); *σ*_*x*_(*i*) is the standard deviation of *P*_*x*_(*i*); *σ*_*y*_(*j*) is the standard deviation of *P*_*y*_(*j*). *σ* represents the filter value applied, which could be 0, 1.0, 1.5, 2.0 and 2.5. α represents the considered direction, which could be 0°, 45°, 90°, and 135°. β represents the top percentage of the histogram curve, which could be 50%, 25%, and 10%.

**Table 2:** Characteristics of the patients in the primary cohort and validation cohort.

Characteristics	Primary cohort		P	Validation cohort		P
	Benign	Malignant		Benign	Malignant	
Age (yr, mean ± SD)	49.60 ± 12.94	62.78 ± 11.59	<0.001*	50.43 ± 14.55	59.77 ± 11.42	<0.001*
Gender						
Male	17 (56.7%)	54 (60%)	0.745	17 (56.7%)	49 (54.4%)	0.832
Female	13 (43.3%)	36 (40%)		13 (43.3%)	41 (45.6%)	
Score (Median[IQR])						
Group 1	−0.091 (−1.170, 1.052)	2.004 (1.155, 2.448)	<0.001*	0.250 (−0.713, 1.449)	1.776 (0.938, 2.248)	<0.001*
Group 2	0.374 (−1.031, 1.273)	1.870 (1.218, 2.341)	<0.001*	0.508 (−0.717, 1.526)	1.606 (0.916, 2.339)	<0.001*
Group 3	0.393 (−0.254, 1.307)	1.548 (1.177, 2.180)	<0.001*	0.458 (−0.607, 1.634)	1.620 (1.096, 1.966)	<0.001*
Group 4	0.636 (−0.190, 1.390)	1.474 (1.046, 1.897)	<0.001*	0.622 (−0.064, 1.603)	1.358 (1.102, 1.746)	<0.001*

Note: IQR = inter-quartile range; Group 1 = non-contrast + 1.25 mm + standard convolution kernel; Group 2 = contrast enhancement + 1.25 mm + standard convolution kernel; Group 3 = non-contrast + 5 mm + standard convolution kernel; Group 4 = non-contrast + 5 mm + lung convolution kernel. p-value is derived from the univariable association analyses between each of the clinicopahological variables and the SPN status. “*” indicates a p-value less than 0.05.

**Table 3:** Diagnostic performance of discrimination and classification of radiomics signature.

Group	Primary cohort							Validation cohort		
	AUC	95%CI	SEN	SPE	Accuracy	AUC	95%CI	SEN	SPE	Accuracy
1	0.862	0.847–0.877	0.944	0.633	0.858	0.750	0.728–0.772	0.922	0.567	0.833
2	0.829	0.813–0.845	0.667	0.867	0.708	0.735	0.715–0.757	0.867	0.533	0.783
3	0.785	0.765–0.805	0.889	0.667	0.825	0.725	0.703–0.747	0.878	0.567	0.800
4	0.770	0.749–0.791	0.878	0.667	0.817	0.686	0.663–0.709	0.822	0.600	0.767

Note: 95%CI: 95% confidence interval. AUC: area under curve. SEN: sensitivity; SPE: specificity. Group 1 = non-contrast + 1.25 mm + standard convolution kernel; Group 2 = contrast enhancement + 1.25 mm + standard convolution kernel; Group 3 = non-contrast + 5 mm + standard convolution kernel; Group 4 = non-contrast + 5 mm + lung convolution kernel.

**Table 4:** NRI of inter-group comparison for the primary cohort and validation cohort.

Groups	Primary cohort				Validation cohort	
	NRI Events	Non-NRI Events	Total NRI Events	NRI Events	Non-NRI Events	Total NRI Events
1 vs. 2	0.244	0.333	0.578	0.022	0.001	0.023
1 vs. 3	0.333	0.533	0.867	0.200	0.267	0.467
3 vs. 4	0.089	0.067	0.156	0.267	0.200	0.467

Note: NRI = Net Reclassification Improvement; NRI Events = Net Reclassification Improvement for events; Non-NRI Events = Net Reclassification Improvement for non-events. Group 1 = non-contrast + 1.25 mm + standard convolution kernel; Group 2 = contrast enhancement + 1.25 mm + standard convolution kernel; Group 3 = non-contrast + 5 mm + standard convolution kernel; Group 4 = non-contrast + 5 mm + lung convolution kernel.
