# Supplementary material for: Effects of contrast-enhancement, reconstruction slice thickness and convolution kernel on the diagnostic performance of radiomics signature in solitary pulmonary nodule
Source: Sci Rep. 2016 Oct 10;6:34921. doi: 10.1038/srep34921 (PMC5056507; doi:10.1038/srep34921)
Supplement: Supplementary Information [file srep34921-s1.pdf]

Supplementary information for:

Effects of contrast-enhancement, reconstruction slice thickness and convolution kernel on the diagnostic performance of radiomics signature in solitary pulmonary nodule

Lan He[1,2+], Yanqi Huang[1+], Zelan Ma[1], Cuishan Liang[1], Changhong Liang[1*], Zaiyi Liu[1*]

[1]Department of Radiology, Guangdong General Hospital, Guangdong Academy of Medical Sciences, Guangzhou, Guangdong, 510080, China,

[2]School of Medicine, South China University of Technology, Guangzhou, Guangdong, 510006, China.

Correspondence and requests for materials should be addressed to Z.L. (zyliu@163.com) or C.L. (cjr.lchh@vip.163.com)

Other authods' Email-addresses are as follows: L.H. (helan0811@126.com); Y.H.(yikiann@126.com);Z.M.(zelanma@163.com);C.L.(405821317@qq.com).

[+]these authors contributed equally to this work

This work was supported by the National Natural Scientific Foundation of China (No. 81271569 and No.81271654)

Supplementary Method S1: The algorithm for radiomics features calculation

The features included gray-level histogram features and gray-level co-occurrence matrix features. The characteristics of gray-level histogram features were quantified using first-order statistics, calculated from the histogram of all tumor pixel intensity values. Gray-level co-occurrence matrix features are available to quantify intra-tumor heterogeneity differences within the tumor. The features of gray-level histogram and gray-level co-occurrence matrix were extracted from the CT image without / after a filtration of the Laplacian of Gaussian filter (filter parameter = 1.0, 1.5, 2.0, 2.5, respectively).

1. *Laplacian of Gaussian filtration for gray-level histogram features and gray-level co-occurrence matrix features*

The Laplacian of Gaussian filter ($\nabla^2 G$) distribution is given by

$$\nabla^2 G(x, y) = \frac{-1}{\pi\sigma^4}(1 - \frac{x^2 + y^2}{2\sigma^2})e^{-(\frac{x^2+y^2}{2\sigma^2})}$$

x, y denote the spatial coordinates of the pixel and σ is the value of filter parameter.

2. *Gray-level histogram features:*

$X(i)$ indicates the intensity of gray level i, N denotes the sum of pixels in the image. β indicates the top percentage of the histogram curve, which could be 50%, 25%, and 10%, M denotes the number of pixels in the histogram on the percentage of $(1 - \beta)$.

1) Mean

$$mean = \frac{1}{N}\sum_{i=1}^{N} X(i)$$

2) SD

$$SD = \frac{1}{N}\sum_{i=1}^{N} (X(i) - \bar{X})^2$$

3) Percentile mean and Percentile SD

$$mean_\beta = \frac{1}{N-M}\sum_{i=M}^{N} X(i)$$

$$SD_\beta = \frac{1}{N-M}\sum_{i=M}^{N} (X(i) - \bar{X})^2$$

4) Kurtosis

$$kurtosis = \frac{\frac{1}{N}\sum_{i=1}^{N}(X(i) - \bar{X})^4}{(\sqrt{\frac{1}{N}\sum_{i=1}^{N}(X(i) - \bar{X})^2})^4}$$

5) Skewness

$$skewness = \frac{\frac{1}{N}\sum_{i=1}^{N}(X(i) - \bar{X})^3}{(\sqrt{\frac{1}{N}\sum_{i=1}^{N}(X(i) - \bar{X})^2})^3}$$

3. Gray-level co-occurrence matrix features:

Gray-level co-occurrence matrix is second-order statistical texture feature, which is defined as a matrix $P(i, j)$ to indicate the relative frequency with intensity values of two pixels (i and j) at the one distances (δ=1) and in four directions (0°, 45°, 90°, 135°). N_g is the number of discrete intensity levels in the image. x, y denote the spatial coordinates of the pixel. μ, $\mu_x(i)$, $\mu_y(j)$ is the mean of $P(i, j)$, $P_x(i)$, $P_y(j)$, and $\sigma_x(i)$, $\sigma_y(j)$is the standard deviation of $P_x(i)$, $P_y(j)$, respectively. Texture matrixes were determined considering 5×5

matrixes. In this study, distance δ was set to 1 and direction to each of the 4 directions, yielding a total of 4 gray level co-occurrence matrices for each image. From these gray-level co-occurrence matrices, several textures features are derived.

1) Contrast

$$contrast = \sum_{i=1}^{N_g} \sum_{j=1}^{N_g} |i-j|^2 \, P(i,j)$$

2) Correlation

$$correlation = \frac{\sum_{i=1}^{N_g} \sum_{j=1}^{N_g} ijP(i,j) - \mu_i(i)\mu_j(j)}{\sigma_x(i)\sigma_y(j)}$$

3) Entropy

$$entropy = -\sum_{i=1}^{N_g} \sum_{j=1}^{N_g} P(i,j) \log[P(i,j)]$$

4) Energy

$$energy = \sum_{i=1}^{N_g} \sum_{j=1}^{N_g} [P(i,j)]^2$$

5) Homogeneity

$$homogeneity = \sum_{i=1}^{N_g} \sum_{j=1}^{N_g} \frac{P(i,j)}{1+|i-j|^2}$$

Supplementary Equations S1

$$\text{Score} = -1.78771807 + 13.212427\times\text{homogeneity\_90\_0} + 18.54771\times\text{entropy\_135\_0}$$
$$+ 1.0421137000\times\text{entropy\_135\_1.0} - 0.002951890\times\text{his\_50\_mean\_1.0}$$
$$+ 0.0053501100\times\text{his\_25\_mean\_1.0} - 0.0000172400\times\text{his\_mean\_0}$$
$$+ 9.09550720\times\text{homogeneity\_90\_1.5} - 0.00171663\times\text{his\_mean\_2.0}$$
$$- 7.87378419\times\text{energy\_135\_1.5} - 13.14641130\times\text{homogeneity\_45\_1.5}$$
$$- 29.415270830\times\text{homogeneity\_90\_2.5} - 13.27280731\times\text{entropy\_0\_2.5}$$

Supplementary Equations S2

$$\text{Score} = -22.511849 - 40.800501\times\text{energy\_45\_0} - 2.461693\times\text{skewness\_1.0}$$
$$+ 12.073593\times\text{entropy\_45\_1.0} - 5.159926\times\text{homogeneity\_135\_2.0}$$

Supplementary Equations S3

$$\text{Score} = 18.129660 - 51.721882\times\text{energy\_45\_1.0} - 88.518050\times\text{energy\_135\_1.0}$$
$$- 8.326476\times\text{homogeneity\_90\_1.0}$$

Supplementary Equations S4

$$\text{Score} = -24.131532 + 6.607335\times\text{entropy\_45\_0} + 5.495184\times\text{entropy\_45\_1.0} - 9.753482\times \text{homogeneity\_90\_2.0}$$

Supplementary Table S1. The size and the diameter of the delineations of ROI for 2 patients who have benign and malignant tumor, respectively.

Group	Benign		Malignant	
	Size	Diameter	Size	Diameter
1	792.330	35.155	208.603	15.871
2	780.833	33.084	212.640	16.610
3	782.832	35.155	206.584	16.980
4	790.331	34.438	209.948	16.536

Note: Group 1 = non-contrast + 1.25 mm + standard convolution kernel; Group 2 = contrast enhancement + 1.25 mm + standard convolution kernel; Group 3 = non-contrast + 5 mm + standard convolution kernel; Group 4 = non-contrast + 5 mm + lung convolution kernel.

Supplementary Table S2. Intra-class correlation coefficients (ICCs) values for radiomics features

Radiomics features	Group 1					Group 2					Group 3					Group 4				
	**0**	**1.0**	**1.5**	**2.0**	**2.5**	**0**	**1.0**	**1.5**	**2.0**	**2.5**	**0**	**1.0**	**1.5**	**2.0**	**2.5**	**0**	**1.0**	**1.5**	**2.0**	**2.5**
**Gray-level**																				
kurtosis	0.913	0.960	0.892	0.855	0.833	0.962	0.932	0.961	0.962	0.967	0.986	0.972	0.988	0.990	0.990	0.982	0.964	0.983	0.988	0.989
skewness	0.907	0.843	0.882	0.832	0.817	0.878	0.878	0.880	0.906	0.912	0.871	0.841	0.889	0.922	0.931	0.981	0.914	0.879	0.905	0.914
his_mean	0.995	0.995	0.995	0.995	0.995	0.998	0.998	0.998	0.998	0.998	0.997	0.997	0.997	0.997	0.997	0.993	0.993	0.993	0.993	0.993
his_SD	0.995	0.993	0.990	0.995	0.990	0.991	0.996	0.992	0.994	0.998	0.994	0.999	0.994	0.993	0.995	0.998	0.992	0.998	0.996	0.998
his_50_mean	0.997	0.997	0.998	0.997	0.997	0.999	0.999	0.999	0.999	0.999	0.999	0.999	0.999	0.999	0.999	0.998	0.983	0.998	0.998	0.998
his_50_SD	0.994	0.993	0.989	0.994	0.990	0.990	0.996	0.991	0.993	0.997	0.994	0.999	0.993	0.992	0.995	0.997	0.990	0.997	0.995	0.998
his_25_mean	0.916	0.801	0.809	0.975	0.984	0.999	0.816	0.999	0.999	0.999	1.000	0.858	1	1.000	0.991	0.998	0.757	0.999	0.999	0.999
his_25_SD	0.935	0.750	0.616	0.981	0.989	0.987	0.902	0.989	0.992	0.997	0.992	0.749	0.991	0.991	0.993	0.997	0.841	0.997	0.994	0.998
his_10_mean	0.992	0.836	0.996	0.993	0.995	0.993	0.881	0.986	0.997	0.986	0.989	0.879	0.942	1.000	0.999	0.943	0.778	0.819	0.992	0.980
his_10_SD	0.999	0.869	0.999	0.999	1	0.981	0.763	0.981	0.987	0.997	0.986	0.798	0.974	0.985	0.990	0.994	0.790	0.762	0.987	0.994
**Gray-Level Co-Occurrence Matrix (GLCM)**																				
Contrast																				
0	0.989	0.975	0.966	0.970	0.978	0.996	0.991	0.992	0.991	0.992	0.995	0.976	0.990	0.986	0.983	0.993	0.984	0.989	0.989	0.988
45	0.926	0.989	0.856	0.750	0.710	0.994	0.998	0.993	0.993	0.993	0.988	0.976	0.983	0.977	0.973	0.988	0.979	0.977	0.965	0.965
90	0.978	0.988	0.966	0.961	0.964	0.992	0.990	0.987	0.986	0.987	0.995	0.991	0.990	0.981	0.976	0.993	0.991	0.991	0.987	0.987
135	0.962	0.974	0.842	0.752	0.757	0.990	0.987	0.983	0.977	0.975	0.994	0.992	0.985	0.968	0.961	0.988	0.985	0.981	0.975	0.975
Correlation																				
0	0.989	0.991	0.981	0.968	0.948	0.996	0.995	0.994	0.994	0.993	0.996	0.977	0.992	0.988	0.986	0.994	0.989	0.991	0.992	0.991

45	0.950	0.934	0.919	0.913	0.908	0.991	0.987	0.992	0.995	0.995	0.994	0.969	0.993	0.988	0.988	0.994	0.989	0.994	0.991	0.991
90	0.978	0.968	0.960	0.967	0.971	0.992	0.990	0.989	0.991	0.990	0.996	0.994	0.995	0.992	0.988	0.993	0.991	0.993	0.991	0.991
135	0.987	0.994	0.981	0.983	0.983	0.992	0.991	0.994	0.992	0.990	0.998	0.994	0.998	0.993	0.989	0.991	0.988	0.989	0.990	0.990
Entropy																				
0	0.998	0.941	0.995	0.981	0.975	0.995	0.987	0.992	0.989	0.988	0.991	0.989	0.989	0.992	0.992	0.995	0.994	0.992	0.990	0.991
45	0.995	0.888	0.995	0.969	0.958	0.993	0.985	0.991	0.988	0.988	0.992	0.984	0.991	0.995	0.993	0.994	0.989	0.992	0.991	0.993
90	0.993	0.953	0.992	0.987	0.970	0.996	0.990	0.994	0.992	0.991	0.991	0.987	0.990	0.991	0.992	0.995	0.991	0.992	0.993	0.995
135	0.993	0.966	0.987	0.969	0.958	0.996	0.988	0.994	0.993	0.993	0.988	0.986	0.988	0.992	0.993	0.995	0.993	0.993	0.994	0.995
Energy																				
0	0.998	0.939	0.996	0.979	0.972	0.995	0.990	0.992	0.989	0.989	0.991	0.992	0.990	0.993	0.993	0.996	0.997	0.992	0.992	0.994
45	0.995	0.793	0.995	0.974	0.966	0.995	0.992	0.992	0.990	0.990	0.994	0.991	0.993	0.995	0.994	0.997	0.995	0.995	0.995	0.995
90	0.992	0.928	0.996	0.982	0.965	0.996	0.993	0.994	0.991	0.991	0.991	0.991	0.992	0.992	0.992	0.996	0.994	0.993	0.994	0.995
135	0.997	0.958	0.995	0.969	0.962	0.996	0.993	0.994	0.993	0.993	0.990	0.991	0.991	0.993	0.993	0.995	0.996	0.993	0.995	0.996
Homogeneity																				
0	0.986	0.996	0.951	0.888	0.854	0.991	0.985	0.982	0.980	0.980	0.986	0.974	0.980	0.977	0.975	0.992	0.981	0.988	0.988	0.987
45	0.915	0.868	0.930	0.921	0.889	0.992	0.984	0.982	0.976	0.979	0.978	0.975	0.970	0.965	0.963	0.986	0.972	0.979	0.965	0.963
90	0.962	0.919	0.953	0.968	0.973	0.993	0.990	0.987	0.980	0.980	0.992	0.980	0.985	0.978	0.975	0.992	0.987	0.987	0.983	0.982
135	0.885	0.980	0.785	0.652	0.785	0.992	0.984	0..988	0.974	0.970	0.970	0.985	0.957	0.959	0.965	0.982	0.968	0.970	0.948	0.937

Note： Group 1 = non-contrast + 1.25 mm + standard convolution kernel; Group 2 = contrast enhancement + 1.25 mm + standard convolution kernel; Group 3 = non-contrast + 5 mm + standard convolution kernel; Group 4 = non-contrast + 5 mm + lung convolution kernel.

Supplementary Table S3. Univariate association of radiomics features with the status of SPN in the primary and validation cohort

Group 1					Group 2					Group 3					Group 4				
diomics features	Primary cohort		Validation cohort		radiomics features	Primary cohort		Validation cohort		radiomics features	Primary cohort		Validation cohort		radiomics features	Primary cohort		Validation cohort	
	AUC	P	AUC	P		AUC	P	AUC	P		AUC	P	AUC	P		AUC	P	AUC	P
ergy_0_0	0.784	<0.001	0.750	<0.001	energy_0_0	0.729	<0.001	0.763	<0.001	energy_90_0	0.694	<0.001	0.657	0.010	energy_45_0	0.736	<0.001	0.688	0.002
ergy_45_0	0.791	<0.001	0.755	<0.001	energy_45_0	0.776	<0.001	0.689	0.002	energy_135_0	0.720	0.001	0.654	0.012	energy_90_0	0.727	<0.001	0.696	0.001
ergy_90_0	0.790	<0.001	0.756	<0.001	energy_90_0	0.728	<0.001	0.767	<0.001	homogeneity_135_0	0.702	0.003	0.630	0.033	energy_135_0	0.737	<0.001	0.711	<0.001
ergy_135_0	0.791	<0.001	0.756	<0.001	energy_135_0	0.721	<0.001	0.779	<0.001	entropy_0_0	0.680	<0.001	0.690	0.002	homogeneity_45_0	0.674	0.004	0.643	0.019
mogeneity_0_0	0.692	0.002	0.635	0.027	homogeneity_0_0	0.644	0.019	0.657	0.010	entropy_45_0	0.721	<0.001	0.659	0.009	homogeneity_90_0	0.672	0.005	0.633	0.030
mogeneity_45_0	0.738	<0.001	0.657	0.010	homogeneity_45_0	0.671	0.005	0.622	0.046	entropy_90_0	0.728	<0.001	0.690	0.008	homogeneity_135_0	0.659	0.009	0.674	0.004
mogeneity_90_0	0.710	<0.001	0.646	0.017	homogeneity_135_0	0.675	0.004	0.696	0.001	entropy_135_0	0.749	<0.001	0.689	0.020	entropy_0_0	0.726	<0.001	0.709	<0.001
mogeneity_135_0	0.719	<0.001	0.710	0.001	entropy_0_0	0.716	<0.001	0.739	<0.001	his_mean_0	0.729	0.013	0.662	0.037	entropy_45_0	0.744	<0.001	0.693	0.002
tropy_0_0	0.771	<0.001	0.732	<0.001	entropy_45_0	0.733	<0.001	0.753	<0.001	his_50_mean_0	0.652	0.023	0.642	0.006	entropy_90_0	0.729	<0.001	0.701	0.001
tropy_45_0	0.784	<0.001	0.739	<0.001	entropy_90_0	0.722	<0.001	0.745	<0.001	his_25_mean_0	0.639	0.013	0.628	0.012	entropy_135_0	0.744	<0.001	0.719	<0.001
tropy_90_0	0.779	<0.001	0.739	<0.001	entropy_135_0	0.721	<0.001	0.767	<0.001	his_10_mean_0	0.652	0.033	0.668	<0.001	his_mean_0	0.646	0.017	0.690	0.002
tropy_135_0	0.786	<0.001	0.753	<0.001	skewness_1.0	0.717	<0.001	0.644	0.019	his_10_SD_0	0.630	0.035	0.654	0.002	his_25_mean_0	0.650	0.014	0.655	0.011
_mean_0	0.632	0.030	0.656	0.011	energy_0_1.0	0.715	<0.001	0.694	0.001	energy_0_1.0	0.629	<0.001	0.729	<0.001	his_25_SD_0	0.623	0.044	0.640	0.022
_25_mean_0	0.653	0.010	0.644	0.018	energy_45_1.0	0.737	<0.001	0.707	<0.001	energy_45_1.0	0.761	<0.001	0.693	<0.001	energy_0_1.0	0.712	<0.001	0.705	<0.001
ergy_0_1.0	0.727	<0.001	0.673	0.005	energy_90_1.0	0.715	<0.001	0.690	0.002	energy_90_1.0	0.769	<0.001	0.732	<0.001	energy_45_1.0	0.740	<0.001	0.702	<0.001
ergy_45_1.0	0.757	<0.001	0.695	0.001	energy_135_1.0	0.703	<0.001	0.721	<0.001	energy_135_1.0	0.764	<0.001	0.748	<0.001	energy_90_1.0	0.739	<0.001	0.695	0.001
ergy_90_1.0	0.765	<0.001	0.699	0.001	entropy_0_1.0	0.714	<0.001	0.707	0.001	homogeneity_90_1.0	0.784	<0.001	0.621	0.049	energy_135_1.0	0.695	0.001	0.713	<0.001
ergy_135_1.0	0.761	<0.001	0.703	<0.001	entropy_45_1.0	0.732	<0.001	0.717	<0.001	homogeneity_135_1.0	0.733	0.004	0.668	0.006	entropy_0_1.0	0.732	<0.001	0.711	<0.001
mogeneity_90_1.0	0.693	0.002	0.648	0.015	entropy_90_1.0	0.710	<0.001	0.692	0.002	entropy_0_1.0	0.674	<0.001	0.737	<0.001	entropy_45_1.0	0.751	<0.001	0.707	0.001
tropy_0_1.0	0.727	<0.001	0.693	0.002	entropy_135_1.0	0.691	0.002	0.732	<0.001	entropy_45_1.0	0.748	<0.001	0.708	0.001	entropy_90_1.0	0.753	<0.001	0.699	0.001
tropy_45_1.0	0.757	<0.001	0.706	<0.001	kurtosis_1.5	0.673	0.005	0.701	0.001	entropy_90_1.0	0.767	<0.001	0.740	<0.001	entropy_135_1.0	0.706	0.001	0.716	<0.001

tropy_90_1.0	0.765	<0.001	0.706	0.001	energy_0_1.5	0.729	<0.001	0.766	<0.001	entropy_135_1.0	0.756	<0.001	0.747	<0.001	his_mean_1.0	0.646	0.017	0.690	0.002
tropy_135_1.0	0.761	<0.001	0.722	<0.001	energy_45_1.5	0.727	<0.001	0.757	<0.001	his_mean_1.0	0.774	0.013	0.662	0.008	energy_0_1.5	0.700	0.001	0.710	<0.001
_mean_1.0	0.632	0.031	0.656	0.011	energy_90_1.5	0.727	<0.001	0.773	<0.001	his_50_mean_1.0	0.652	0.022	0.646	0.017	energy_45_1.5	0.730	<0.001	0.704	0.001
_50_mean_1.0	0.620	0.049	0.640	0.022	energy_135_1.5	0.721	<0.001	0.780	<0.001	his_25_SD_1.0	0.640	0.003	0.628	0.036	energy_90_1.5	0.728	<0.001	0.703	0.001
_25_mean_1.0	0.623	0.044	0.637	0.026	homogeneity_90_1.5	0.674	0.004	0.631	0.032	entropy_0_1.5	0.684	0.003	0.652	0.013	energy_135_1.5	0.715	<0.001	0.729	<0.001
_25_SD_1.0	0.657	0.010	0.686	0.002	homogeneity_135_1.5	0.687	0.002	0.709	<0.001	entropy_90_1.5	0.683	<0.001	0.658	0.010	homogeneity_45_1.5	0.681	0.003	0.623	0.045
rtosis_1.5	0.703	0.001	0.699	0.001	entropy_0_1.5	0.723	<0.001	0.738	<0.001	entropy_135_1.5	0.721	0.001	0.644	0.018	homogeneity_90_1.5	0.740	<0.001	0.641	0.021
ergy_0_1.5	0.769	<0.001	0.773	<0.001	entropy_45_1.5	0.736	<0.001	0.746	<0.001	his_mean_1.5	0.695	0.013	0.662	0.008	homogeneity_135_1.5	0.675	0.004	0.661	0.008
ergy_45_1.5	0.771	<0.001	0.769	<0.001	entropy_90_1.5	0.713	<0.001	0.748	<0.001	his_50_mean_1.5	0.652	0.017	0.642	0.021	entropy_0_1.5	0.707	<0.001	0.708	<0.001
ergy_90_1.5	0.779	<0.001	0.781	<0.001	entropy_135_1.5	0.717	<0.001	0.761	<0.001	his_25_mean_1.5	0.646	0.015	0.627	0.038	entropy_45_1.5	0.747	<0.001	0.703	<0.001
ergy_135_1.5	0.776	<0.001	0.776	<0.001	kurtosis_2.0	0.644	0.018	0.702	<0.001	his_mean_2.0	0.649	0.013	0.662	0.008	entropy_90_1.5	0.733	<0.001	0.701	<0.001
mogeneity_45_1.5	0.743	<0.001	0.654	0.012	energy_0_2.0	0.723	<0.001	0.763	<0.001	his_50_mean_2.0	0.652	0.044	0.642	0.020	entropy_135_1.5	0.719	<0.001	0.726	<0.001
mogeneity_90_1.5	0.716	<0.001	0.663	0.008	energy_45_2.0	0.716	<0.001	0.761	<0.001	his_25_mean_2.0	0.624	0.016	0.630	0.034	his_mean_1.5	0.646	0.017	0.690	0.002
mogeneity_135_1.5	0.699	0.001	0.686	0.002	energy_90_2.0	0.720	<0.001	0.777	<0.001	his_10_mean_2.0	0.647	0.023	0.624	0.042	his_25_mean_1.5	0.647	0.016	0.656	0.011
tropy_0_1.5	0.754	<0.001	0.733	<0.001	energy_135_2.0	0.711	<0.001	0.772	<0.001	his_mean_2.5	0.639	0.013	0.662	0.008	his_25_SD_1.5	0.627	0.037	0.635	0.028
tropy_45_1.5	0.767	<0.001	0.754	<0.001	homogeneity_135_2.0	0.689	0.002	0.712	0.001	his_SD_2.5	0.622	0.046	0.620	0.049	his_10_mean_1.5	0.626	0.039	0.687	0.002
tropy_90_1.5	0.764	<0.001	0.743	<0.001	entropy_0_2.0	0.718	<0.001	0.737	<0.001	his_50_mean_2.5	0.622	0.047	0.644	0.019	his_10_SD_1.5	0.633	0.029	0.689	0.002
tropy_135_1.5	0.769	<0.001	0.752	<0.001	entropy_45_2.0	0.721	<0.001	0.733	<0.001	his_25_mean_2.5	0.649	0.015	0.628	0.037	energy_0_2.0	0.667	0.006	0.663	0.008
_mean_1.5	0.632	0.031	0.656	0.011	entropy_90_2.0	0.722	<0.001	0.746	<0.001						energy_45_2.0	0.686	0.002	0.651	0.013
_25_mean_1.5	0.654	0.012	0.647	0.017	entropy_135_2.0	0.715	<0.001	0.754	<0.001						energy_90_2.0	0.701	0.001	0.670	0.005
ergy_0_2.0	0.716	<0.001	0.743	<0.001	his_25_mean_2.0	0.631	0.032	0.630	0.034						energy_135_2.0	0.679	0.003	0.671	0.005
ergy_45_2.0	0.722	<0.001	0.742	<0.001	energy_0_2.5	0.702	<0.001	0.750	<0.001						homogeneity_90_2.0	0.768	<0.001	0.649	0.015
ergy_90_2.0	0.731	<0.001	0.763	<0.001	energy_45_2.5	0.670	0.001	0.751	<0.001						entropy_0_2.0	0.690	0.002	0.680	0.003
ergy_135_2.0	0.709	<0.001	0.750	<0.001	energy_90_2.5	0.702	<0.001	0.760	<0.001						entropy_45_2.0	0.721	<0.001	0.669	0.006
mogeneity_45_2.0	0.667	0.006	0.623	0.044	energy_135_2.5	0.695	0.001	0.755	<0.001						entropy_90_2.0	0.727	<0.001	0.677	0.004
mogeneity_90_2.0	0.717	<0.001	0.693	0.002	homogeneity_135_2.5	0.668	0.006	0.693	0.002						entropy_135_2.0	0.700	0.001	0.677	0.004

mogeneity_135_2.0	0.627	0.037	0.681	0.003	entropy_0_2.5	0.690	0.002	0.732	<0.001	his_mean_2.0	0.646	0.017	0.690	0.002
tropy_0_2.0	0.728	<0.001	0.737	<0.001	entropy_45_2.5	0.703	<0.001	0.718	<0.001	his_25_mean_2.0	0.651	0.013	0.638	0.024
tropy_45_2.0	0.741	<0.001	0.744	<0.001	entropy_90_2.5	0.707	<0.001	0.736	<0.001	his_10_mean_2.0	0.628	0.036	0.671	0.005
tropy_90_2.0	0.750	<0.001	0.752	<0.001	entropy_135_2.5	0.700	0.001	0.736	<0.001	energy_0_2.5	0.632	0.031	0.645	0.018
tropy_135_2.0	0.722	<0.001	0.746	<0.001	his_25_mean_2.5	0.632	0.031	0.622	0.046	energy_45_2.5	0.655	0.011	0.626	0.040
_mean_2.0	0.632	0.031	0.656	0.011						energy_90_2.5	0.665	0.007	0.648	0.015
_25_mean_2.0	0.656	0.011	0.626	0.039						energy_135_2.5	0.644	0.018	0.667	0.006
ergy_0_2.5	0.684	0.003	0.728	<0.001						homogeneity_90_2.5	0.756	<0.001	0.630	0.033
ergy_45_2.5	0.685	0.002	0.729	<0.001						entropy_0_2.5	0.660	0.009	0.665	0.007
ergy_90_2.5	0.704	<0.001	0.750	<0.001						entropy_45_2.5	0.696	0.001	0.638	0.024
ergy_135_2.5	0.669	0.006	0.739	<0.001						entropy_90_2.5	0.689	0.002	0.655	0.011
mogeneity_90_2.5	0.710	<0.001	0.682	0.003						entropy_135_2.5	0.662	0.008	0.661	0.008
mogeneity_135_2.5	0.623	0.045	0.664	0.007						his_mean_2.5	0.646	0.017	0.690	0.002
tropy_0_2.5	0.686	0.002	0.727	<0.001						his_25_mean_2.5	0.651	0.013	0.633	0.030
tropy_45_2.5	0.717	<0.001	0.729	<0.001						his_10_mean_2.5	0.620	0.049	0.659	0.009
tropy_90_2.5	0.716	<0.001	0.744	<0.001										
tropy_135_2.5	0.683	0.003	0.729	<0.001										
_mean_2.5	0.632	0.031	0.656	0.011										
_25_mean_2.5	0.655	0.011	0.625	0.042										

Note： Group 1 = non-contrast + 1.25 mm + standard convolution kernel; Group 2 = contrast enhancement + 1.25 mm + standard convolution kernel; Group 3 = non-contrast + 5 mm + standard convolution kernel; Group 4 = non-contrast + 5 mm + lung convolution kernel. **p-**value is derived from the univariable association analyses between each of the features and the SPN status.

Supplementary Table S4. Radiomics features with non-zero coefficient selected in lasso logistic regression

Group 1		Group 2		Group 3		Group 4	
**Radiomics features**	**Coefficient**	**Radiomics features**	**Coefficient**	**Radiomics features**	**Coefficient**	**Radiomics features**	**Coefficient**
homogeneity_90_0	13.212	energy_45_0	-40.801	energy_45_1.0	-51.722	entropy_45_0	6.607
entropy_135_0	18.548	skewness_1.0	-2.462	energy_135_1.0	-88.518	entropy_45_1.0	5.495
his_mean_0	-0.00002	entropy_45_1.0	12.074	homogeneity_90_1.0	-8.326	homogeneity_90_2.0	-9.753
entropy_135_1.0	1.042	homogeneity_135_2.0	-5.160				
his_50_mean_1.0	-0.003						
his_25_mean_1.0	0.005						
energy_135_1.5	-7.874						
homogeneity_45_1.5	-13.146						
homogeneity_90_1.5	9.096						
his_mean_2.0	-0.002						
homogeneity_90_2.5	-29.415						
entropy_0_2.5	-13.273						

Note： Group 1 = non-contrast + 1.25 mm + standard convolution kernel; Group 2 = contrast enhancement + 1.25 mm + standard convolution kernel; Group 3 = non-contrast + 5 mm + standard convolution kernel; Group 4 = non-contrast + 5 mm + lung convolution kernel.

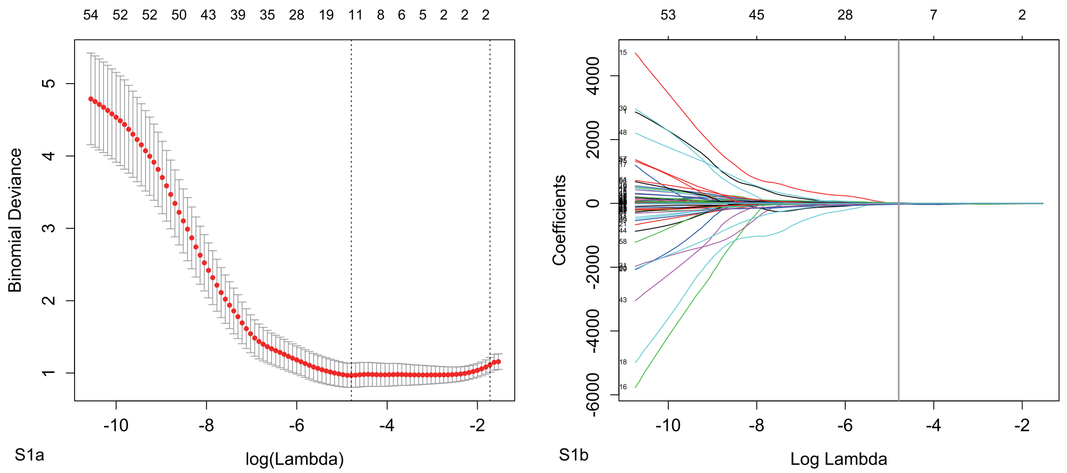

Supplementary Figure S1. Representative diagram for feature selection using the LASSO logistic regression analysis in group 1 (non-contrast + 1.25 mm + standard convolution kernel). Figure S1a indicated tuning parameter (λ) selection in the LASSO model used 10-fold cross-validation via minimum criteria. The binomial deviance curve was plotted versus log(λ). The dotted vertical lines were drawn at the optimal values using the minimum criteria and the 1-SE criteria. A value λ of 0.008 with log (λ)=-4.828 was chosen (minimum criteria) according to 10-fold cross-validation. Figure S1b indicated LASSO coefficient profiles of the 66 radiomics features. A coefficient profile plot was produced against the log-lambda sequence. The vertical line was drawn at the value selected using 10-fold cross-validation, where the optimal λ resulted in 12 non-zero coefficients.

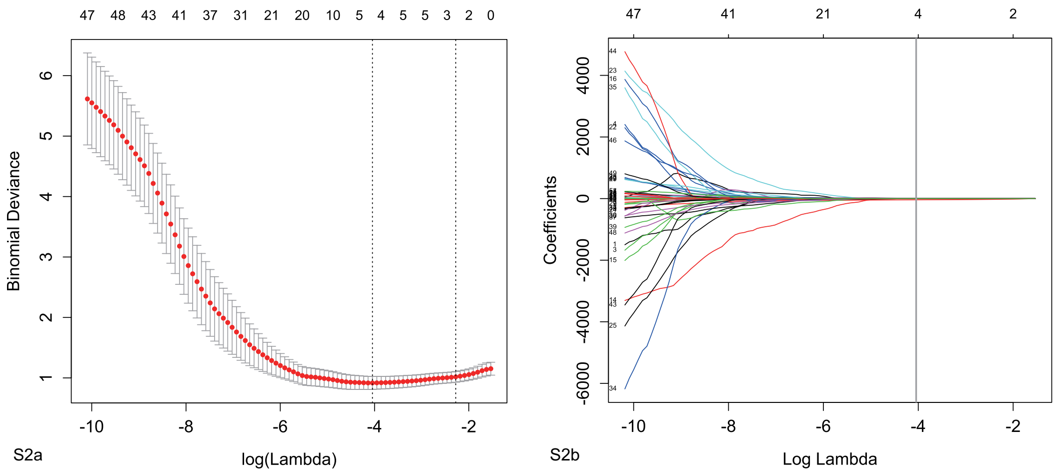

Supplementary Figure S2. Representative diagram for feature selection using the LASSO logistic regression analysis in group 2 (contrast enhancement + 1.25 mm + standard convolution kernel). Figure S2a indicated tuning parameter (λ) selection in the LASSO model used 10-fold cross-validation via minimum criteria. The binomial deviance curve was plotted versus log(λ). The dotted vertical lines were drawn at the optimal values using the minimum criteria and the 1-SE criteria. A value λ of 0.018 with log (λ)=-4.017 was chosen (minimum criteria) according to 10-fold cross-validation. Figure S2b indicated LASSO coefficient profiles of the 52 radiomics features. A coefficient profile plot was produced against the log-lambda sequence. The vertical line was drawn at the value selected using 10-fold cross-validation, where the optimal λ resulted in 4 non-zero coefficients.

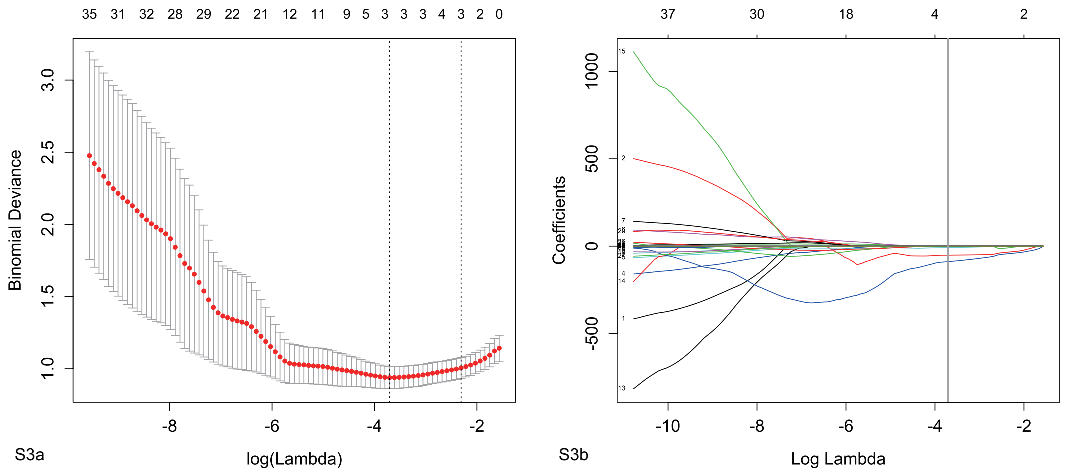

Supplementary Figure S3. Representative diagram for feature selection using the LASSO logistic regression analysis in group 3 (non-contrast + 5 mm + standard convolution kernel). Figure S3a indicated tuning parameter (λ) selection in the LASSO model used 10-fold cross-validation via minimum criteria. The binomial deviance curve was plotted versus log(λ). The dotted vertical lines were drawn at the optimal values using the minimum criteria and the 1-SE criteria. A value λ of 0.025 with log (λ)=-3.689 was chosen (minimum criteria) according to 10-fold cross-validation. Figure S3b indicated LASSO coefficient profiles of the 39 radiomics features. A coefficient profile plot was produced against the log-lambda sequence. The vertical line was drawn at the value selected using 10-fold cross-validation, where the optimal λ resulted in 3 non-zero coefficients.

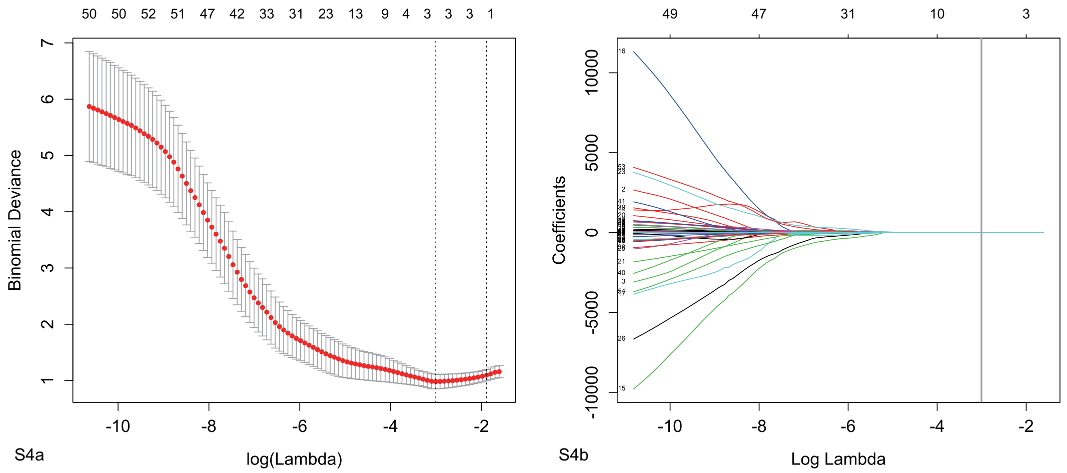

Supplementary Figure S4. Representative diagram for feature selection using the LASSO logistic regression analysis in group 4 (non-contrast + 5 mm + lung convolution kernel). Figure S4a indicated tuning parameter (λ) selection in the LASSO model used 10-fold cross-validation via minimum criteria. The binomial deviance curve was plotted versus log(λ). The dotted vertical lines were drawn at the optimal values using the minimum criteria and the 1-SE criteria. A value λ of 0.050 with log (λ)=-2.996 was chosen (minimum criteria) according to 10-fold cross-validation. Figure S4b indicated LASSO coefficient profiles of the 62 radiomics features. A coefficient profile plot was produced against the log-lambda sequence. The vertical line was drawn at the value selected using 10-fold cross-validation, where the optimal λ resulted in 3 non-zero coefficients.

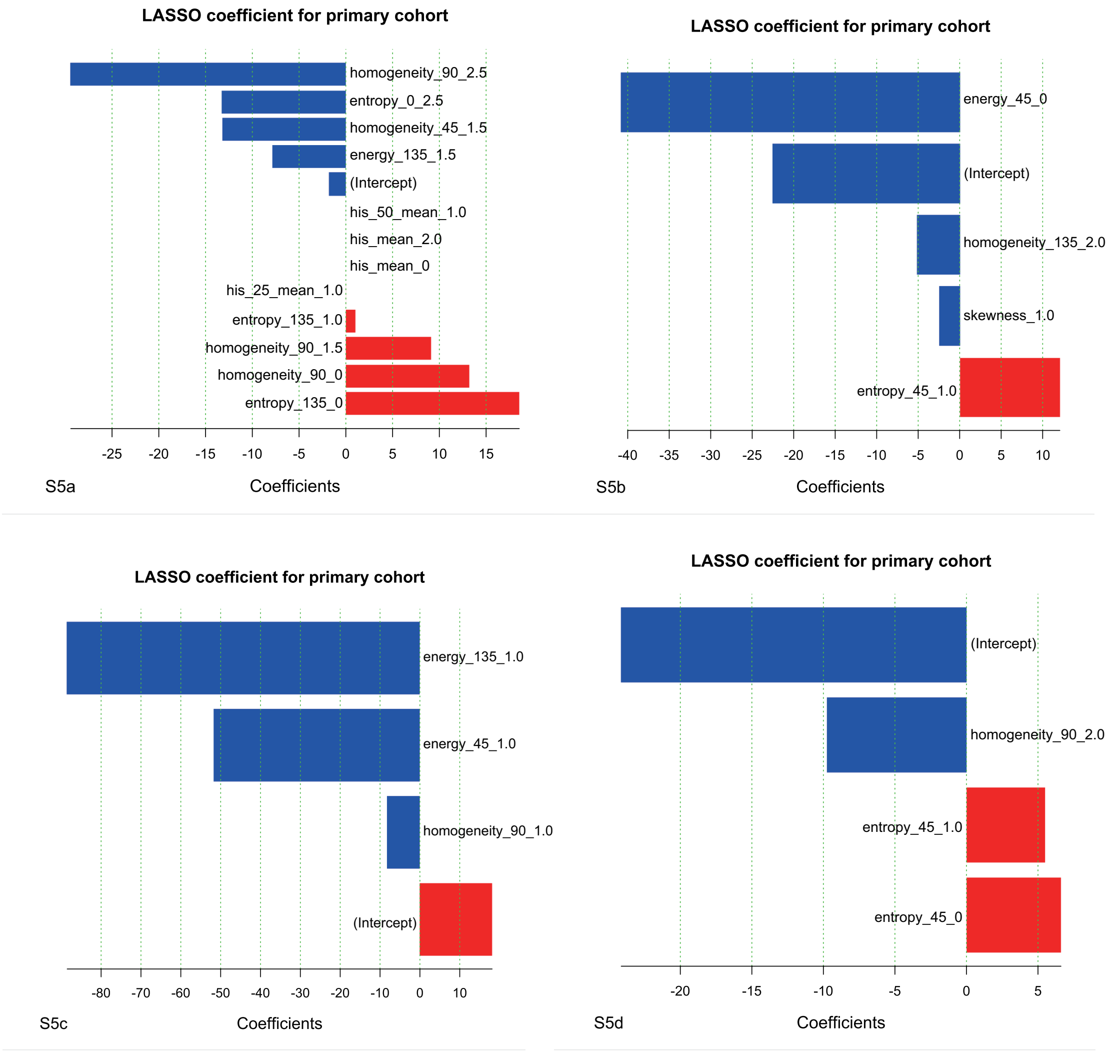

Supplementary Figure S5 Histogram of features selected by non-zero coefficients in the Lasso logistic regression analysis in group 1 (Figure S5a), group 2 (Figure S5b), group 3 (Figure S5c) and group 4 (Figure S5d). Y axis indicated the selected features and X axis indicted the value of the positive (red histogram) or negative (blue histogram) coefficients. 12, 4, 3 and 3 different features were selected in group 1, 2, 3 and 4, respectively.
